# Human Placental LRP5 and Sclerostin are Increased in Gestational Diabetes Mellitus Pregnancies

**DOI:** 10.1210/clinem/dgad164

**Published:** 2023-03-22

**Authors:** Anna Papadopoulou, Eirini Thymara, Eirini Maratou, George Kanellopoulos, Vasiliki Papaevangelou, Sophia Kalantaridou, Spyridon Kanellakis, Pinelopi Triantafyllidou, George Valsamakis, George Mastorakos

**Affiliations:** Third Department of Pediatrics, National and Kapodistrian University of Athens, Medical School, University General Hospital “Attikon,” GR-12464, Athens, Greece; Department of Clinical Biochemistry, National and Kapodistrian University of Athens, Medical School, University General Hospital “Attikon,” GR-12464, Athens, Greece; Department of Pathology, National and Kapodistrian University of Athens, Medical School, GR-11527 Athens, Greece; Department of Pathology, National and Kapodistrian University of Athens, Medical School, GR-11527 Athens, Greece; Third Department of Pediatrics, National and Kapodistrian University of Athens, Medical School, University General Hospital “Attikon,” GR-12464, Athens, Greece; Third Department of Pediatrics, National and Kapodistrian University of Athens, Medical School, University General Hospital “Attikon,” GR-12464, Athens, Greece; Third Department of Obstetrics and Gynecology, National and Kapodistrian University of Athens, Medical School, University General Hospital “Attikon,” Athens, Greece; Department of Nutrition and Dietetics, School of Health Science and Education, Harokopio University, 17676 Athens, Greece; Third Department of Pediatrics, National and Kapodistrian University of Athens, Medical School, University General Hospital “Attikon,” GR-12464, Athens, Greece; Diabetes Mellitus and Metabolism Unit, ARETAION Hospital, Medical School, National and Kapodistrian University of Athens, GR-11528, Athens, Greece; Diabetes Mellitus and Metabolism Unit, ARETAION Hospital, Medical School, National and Kapodistrian University of Athens, GR-11528, Athens, Greece

**Keywords:** sclerostin, LRP5, gestational diabetes mellitus, placenta, umbilical sclerostin

## Abstract

**Introduction:**

The low-density lipoprotein receptor-related protein 5 (LRP5) and its inhibitor sclerostin, are key components of bone metabolism and potential contributors to type 2 diabetes mellitus susceptibility. This study aims at evaluating the expression of placental LRP5 and sclerostin in pregnancies with gestational diabetes mellitus (GDM) and investigate possible associations with umbilical sclerostin concentrations and clinical outcomes in mothers and their neonates.

**Methods:**

Twenty-six GDM-mothers and 34 non-GDM mothers of Caucasian origin and their neonates admitted in a gynecology and obstetrics department of a university hospital were included in this study. Demographic data and maternal fasting glucose concentrations (24-28 weeks of gestation) were retrieved from the patients’ medical records. Placental LRP5 was determined by immunohistochemistry (IHC) and Western blotting analysis; placental sclerostin was determined by IHC. Umbilical serum sclerostin concentrations were measured by ELISA.

**Results:**

Placental sclerostin IHC intensity values were positively correlated with LRP5 values as detected either by IHC (*r* = 0.529; *P <* .001) or Western blotting (*r* = 0.398; *P* = .008), with pregestational maternal body mass index values (*r* = 0.299; *P* = .043) and with maternal fasting glucose concentrations (*r* = 0.475; *P* = .009). Placental sclerostin and LRP5 were significantly greater in GDM compared with non-GDM placentas (histo-score: 65.08 ± 17.09 vs 11.45 ± 2.33, *P <* .001; 145.53 ± 43.74 vs 202.88 ± 58.65, *P <* .001; respectively).

**Discussion:**

Sclerostin and LRP5 were detected in human placentas. The overexpression of placental sclerostin and LRP5 values in GDM compared with non-GDM pregnancies, as well as the positive association of placental sclerostin values with pregestational maternal body mass index and maternal fasting glucose concentrations may indicate the development of an adaptive mechanism in face of maternal hyperglycemia.

The low-density lipoprotein receptor-related protein 5 (LRP5) and its potent antagonist, sclerostin, are start members of the so-called canonical Wnt-signalling pathway, and their action has been mainly associated with the regulation of bone metabolism (1-4). In humans, loss- or gain-of-function mutations in the *LRP5* gene cause low or high bone mass, respectively (5-7). Likewise, progressive bone overgrowth may result from homozygous for loss-of-function mutations in the *SOST* gene encoding for sclerostin (8). Importantly, an antisclerostin antibody, romosozumab, has been recently approved for use in the United States and other countries for increasing bone mass and reducing fracture risk in osteoporosis (9).

Human *LRP5* gene has been mapped within the insulin-dependent diabetes mellitus 4 region on chromosome 11q13 (10). This region is linked to type 1 diabetes mellitus, whereas single nucleotide polymorphisms in *WNT5b* and *LRP5* genes may contribute to susceptibility to type 2 diabetes mellitus (T2DM) and obesity (11-13). Thus, it has been suggested that *LRP5* may be involved in the pathogenesis of T2DM. Later, experiments in Lrp5−/− mice showed that functional LRP5 is indispensable for normal glucose metabolism (14), whereas Leanza et al showed that LRP5 overexpression delays the onset of diabetes in mouse via insulin-independent mechanisms (15). In addition, sclerostin has been found elevated in T2DM rats. Interestingly, this overexpression was associated with increased mRNA levels of LRP5, whereas in humans, serum sclerostin has been found elevated in patients with T2DM as well as in prediabetic conditions (16, 17).

During normal pregnancy, bone metabolism and calcium homeostasis undergo significant changes in maternal environment as an adaptive response to the increasing nutrient requirements of the growing fetus (18). Placenta senses and responds to these changes by altering the secretion of hormones or other signalling molecules, modifying the expression and the activity of channels and receptors and consequently regulating fetal growth. The presence of Wnt ligands and Frizzled G protein-coupled receptors in placenta supports the implication of Wnt signalling pathway in placental and fetal development (19). To date, a few studies have been produced regarding maternal or umbilical sclerostin levels during healthy or pathological pregnancies, whereas there are no data on LRP5 (20-23)

Gestational diabetes mellitus (GDM) is assimilated to pre-T2DM. According to its unanimously accepted definition, it is first diagnosed in the second or third trimester of pregnancy (usually between the 24th and 28th weeks of gestation) in pregnant women who do not suffer from preexisting type 1 diabetes mellitus or T2DM (24). It affects 7% to 13% of pregnant women, depending on the population studied (HAPO study) (25) and may lead to neonatal macrosomia or have adverse outcomes and long-lasting health consequences in the mother and the child (24). Placenta villi derived from pregnant women with GDM exhibit differentially expressed proteins associated with the development of insulin resistance, hypocalcemia, oxidative stress, placental transport capacity, inflammation, and other factors affecting normal fetal growth (26-28).

To investigate whether LRP5 and sclerostin are present in placental tissue and are associated with GDM, we examined the expression of these 2 molecules at the maternal site of placentas of pregnant women with GDM in comparison to placentas of healthy, nondiabetic pregnant women. In addition, the association of their placental expression to maternal glycemia, to their umbilical concentrations as well as to maternal and neonatal anthropometrics, and fetal echographic parameters were evaluated.

## Materials and Methods

### Subjects

**Figure 1.**
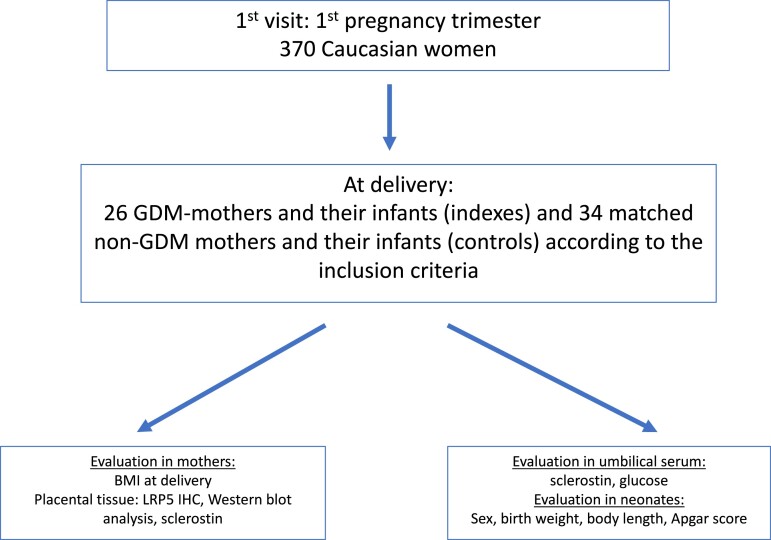
A CONSORT diagram describing the study design.

This is an observational cohort study. A total of 370 primiparous pregnant women of Caucasian origin consulted consecutively during the first trimester of pregnancy at a gynecology and obstetrics department of a university hospital between March 2019 and May 2021. At their first antenatal hospital visit (4-8 gestational weeks [gwk]), maternal demographic and clinical characteristics were recorded, maternal weight and height were measured, and pregestational body mass index (BMI) was calculated (29, 30). On delivery, BMI was calculated again. From these 370 women, 30 were diagnosed with GDM. From them, 26 women and their neonates (indexes) were included in this study according to the following inclusion criteria: no medical history of diabetes; no smoking during the past 6 months; no hypertension during pregnancy; full-term (≥37 gwk) pregnancy; indication to deliver by cesarean section. GDM diagnosis was based on standardized 75 g 2-hour oral glucose tolerance test performed between the 24th and 28th gwk in women according to the HAPO criteria (25). Thirty-four women (without positive diagnosis of GDM at the oral glucose tolerance test performed between the 24th to 28th gwk, non-GDM control women) and their neonates were selected from the 370 initially consulting pregnant women. Control women were matched to GDM pregnant women (indexes) according to their respective pregestational BMI and they satisfied the same inclusion criteria as for women with GDM (Fig. 1). All GDM pregnancies were treated either by diet or insulin. Maternal fasting glucose and insulin concentrations at the 24th to 28th gwk were retrieved from the medical records. As the recruitment of pregnant women took place in the same hospital located in a suburb of Athens, Greece, the selected population was homogeneous regarding place of residence and maternal occupation (almost all women were lower white-collar workers). The study was approved by the institutional review board of the university hospital. Informed consent was obtained from all mothers participating in the study.

### Protocol

At delivery, venous cord blood was collected immediately after clamping. The obtained serum was aliquoted for measurement of sclerostin and glucose, whereas sex, birth weight, body length, and head circumference (HC) of the newborns were obtained by the same neonatologist. The latter was blinded to GDM status. Furthermore, samples of placental tissues were collected from the maternal placenta at a distance of 5 cm from the umbilical cord (cubes of 1 cm/side) immediately after delivery for Western blotting analysis. Tissue biopsies were stored at −80 °C. The rest of the placenta was placed in buffered formalin 10% for further preparation for immunohistochemistry. Measurement of the placental weight was performed on formalin-fixed material after removing extraplacental membranes and the umbilical cord. All specimens for immunohistochemistry were derived from the maternal side of the fixed placenta. Data for fetal femur length, evaluated by echography between 21 to 23 gwk, were gathered from the records (available for 16 and 23 fetuses of GDM and non-GDM pregnancies, respectively).

### Umbilical Sclerostin Measurement

Sclerostin concentrations were measured in umbilical serum by a quantitative sandwich enzyme immunoassay technique (Human Sclerostin [SOST] ELISA Kit Cusabio Biotech, Cat# CSB-E13146h, RRID: AB_2904209) with intra- and interassay coefficient of variation at <8% and <10%, respectively. Detection range was 31.25 pg/mL to 2000pg/mL (1 pg/mL = 0.044 pmol/L).

### Placenta Immunohistochemistry

Four-micrometer placenta sections were deparaffinized in xylene and rehydrated in graded alcohol. Antigen retrieval was performed using 10 mM sodium citrate (pH 6) in a water bath at 95 °C for 15 minutes. Slides were incubated with a rabbit polyclonal anti-LRP5 antibody (Novus, Cat# NBP2-37868, RRID: AB_2904208) at 1:300 dilution or with a rabbit polyclonal anti-human sclerostin/SOST antibody (LSBio (LifeSpan) Cat# LS-C100509-100, RRID: AB_10559962) against an amino acid 134-163 epitope, at 1:50 dilution. Staining was visualized with diaminobenzidine followed by counterstaining with hematoxylin. Tissue sections from the cortical tubular area of human kidney were used as positive controls. Tissue sections in which the primary antibody was substituted with nonimmune serum were used as negative controls. Samples were considered positive if cytoplasmic staining was observed, whereas for microscopic evaluation, only medium and strongly stained cytoplasms or cytoplasmic membrane were considered positive. Samples without membrane staining or without staining were considered negative. Stained sections were examined by 1 investigator (E.T.) who was blinded to the clinical data. Cytoplasmic staining intensity was evaluated according to a semiquantitative scale ranging from 0 (negative) to 1 (mild), 2 (moderate), or 3 (strong). Staining evaluation was performed by a systematic random selection of 10 separate fields of vision at ×20 magnification. In the fields of vision corresponding to a sample, the average of the percentage of LRP5 or sclerostin positive cells (trophoblasts, endothelial cells, cells of the villus stroma, syncytiotrophoblasts) and the intensity of staining in hot spot areas of these cells (areas with at least 100 positive cells) were calculated. Quantitative comparisons between groups of samples were based on histo-score (h-score), which was the percentage of stained cells (labeling index) multiplied by staining intensity.

### Western Blotting

A total of 0.2 to 0.4 g of frozen placental tissues were homogenized using an Ultra turax (IKA labortechnik) (14,000/min) homogenizer in a custom-made ice-cold lysis buffer containing b-mercaptoethanol as reducing reagent and protease and phosphatase inhibitors (#5872S, Cell Signaling USA). The concentration of the proteins was determined using the BCA Protein Assay Kit (#7780S, Cell Signaling, USA). Then, Western blotting for expression levels of the LRP5 and of β-actin, used as internal control, was performed. Proteins were separated in an 8% SDS-PAGE gel (each lane contained 80 μg proteins) and were transferred in a precut nitrocellulose membrane (#1620146, BIORAD). After transfer, the membrane was incubated in blocking buffer (5% nonfat dry milk in PBS-T consisting of 10 mM Tris-HCl [pH 8.0], 150 mM NaCl, and 0.05% [v/v] Tween 20) for 1 hour at room temperature, followed by overnight incubation at 4 °C with the appropriate concentration of anti-LRP5 (Cat # 5731, RRID: AB_10705602), or anti-β-actin (Cat # 4970, AB_223172) rabbit monoclonal antibodies diluted in PBS with Tween (PBST; factor dilution 1:1000). The next day the membrane was washed with PBST for 1 hour and incubated with horseradish peroxidase-conjugated anti-rabbit secondary antibody (#7074, Cell Signalling, USA) diluted at 1:2000 in 5% nonfat dry milk in PBST for another 1 hour at room temperature. The protein was visualized by light emission on film (Fuji X-ray Films RX) and the signal was detected using Clarity Max Western ECL substrate and quantified. Image J system (National Institutes of Health) for the quantification and comparison of the bands was used. The ratio between LRP5 and β-actin signals was calculated for each sample.

### Statistical Analysis

Comparisons between groups were made using Student *t* test or Mann-Whitney test in case of nonparametric variables. Correlations were sought with Pearson correlation coefficient between continuous variables or Spearman correlation coefficient for nonparametric variables. Correlation with type of treatment was performed with linear regression analysis. All hypothesis testing was conducted assuming a 0.05 significance level and a 2-sided alternative hypothesis. Normality of the data was evaluated with Kolmogorov-Smirnov test. Statistical analyses were also performed after adjustment for insulin use, pregestational BMI, and gestational weight gain in the totality of the studied population. For the statistical analysis, we used the IBM SPSS Statistics package, version 24. To our knowledge, there are no data regarding LRP5 and sclerostin in human placentas in the past. Thus, performing a proper power analysis before launching the present study was not feasible.

## Results

### Description and Comparison of Anthropometrics, Type of Treatment, Glucose and Insulin Concentrations in the Study Population

Characteristics of pregnant women and their neonates are summarized in Table 1. Mean age, pregestational BMI and BMI at delivery did not differ between pregnant women with and without GDM. Eight of 26 (30.8%) and 7 of 34 (20.6%) pregnant women with and without GDM, respectively, were overweight, whereas 8 of 26 (30.8%) and 4 of 34 (11.8%) pregnant women with and without GDM, respectively, were obese. Gestational weight gain was significantly greater in pregnant women without GDM compared with those with GDM (mean ± SD, 16.67 ± 8.73 kg and 11.94 ± 5.88 kg, respectively; *P* = .021). Fasting glucose concentrations were greater in pregnant women with GDM compared with those without GDM (mean ± SD, 90.91 ± 20.84 mg/dL and 71.52 ± 9.32 mg/dL, respectively; *P* = .002). Fasting insulin concentrations were higher in pregnant women with GDM compared with those without GDM (mean ± SD, 18.540 ± 8.253 and 12.800 ± 6.675 mU/L, respectively; *P* = .005). Among pregnant women with GDM, 13/26 received insulin treatment and the rest were treated by appropriate diet.

**Table 1.** Anthropometrics and type of treatment in the study population. Baby length, baby head circumference, baby weight, and fetal femur length at 21st through 23rd week of gestation are further analyzed according to maternal pregestational BMI

Mothers	GDM (n = 26)	Non-GDM (n = 34)	*P*
Maternal age, y	33.84 ± 6.15	31.94 ± 5.50	.212
Maternal pregestational BMI, kg/m^2^	27.23 ± 5.26	24.98 ± 4.14	.069
Normal maternal pregestational BMI	22.79 ± 1.31 (Ν = 10; 38.5%)	22.89 ± 1.44 (Ν = 23; 67.6%)	.842
Abnormal maternal pregestational BMI (overweight)	26.55 ± 1.00 (Ν = 8; 30.8%)	26.24 ± 1.20 (Ν = 7; 20.6%)	.589
Abnormal maternal pregestational BMI (obese)	34.31 ± 4.39 (N = 8; 30.8%)	34.84 ± 1.97 (N = 4; 11.8%)	.828
Maternal BMI at delivery, kg/m^2^	31.64 ± 5.24	31.17 ± 4.89	.720
Gestational weight gain, kg	11.94 ± 5.88	16.67 ± 8.73^a^	**.021**
Diet vs insulin controlled GDM	13/13	—	
Infants			
Sex (male-female)	10 boys (40%)	20 (60%)	
Baby length, cm	49.07 ± 4.59	50.06 ± 1.94	.314
Normal maternal pregestational BMI	46.80 ± 6.65 (N = 10; 38.5%)	49.80 ± 2.15 (N = 23; 67.6%)	.193
Abnormal maternal pregestational BMI (overweight)	49.75 ± 1.75 (N = 8; 30.8%)	50.35 ± 1.60 (N = 7; 20.6%)	.498
Abnormal maternal pregestational BMI (obese)	51.29 ± 1.50 (N = 8; 30.8%)	51.00 ± 0.82 (N = 4; 11.8%)	.736
Baby head circumference, cm	36.02 ± 3.49	34.48 ± 1.42^a^	**.043**
Normal maternal pregestational BMI	37.55 ± 5.09 (N = 10; 38.5%)	34.63 ± 1.55^a^ (N = 23; 67.6%)	**.016**
Abnormal maternal pregestational BMI (overweight)	34.75 ± 1.16 (N = 8; 30.8%)	33.78 ± 1.15 (N = 7; 20.6%)	.132
Abnormal maternal pregestational BMI (obese)	35.64 ± 1.75 (N = 8; 30.8%)	34.87 ± 0.63 (N = 4; 11.8%)	.427
Birth weight, g	3231.73 ± 431.74	3149.12 ± 398.56	.451
Normal maternal pregestational BMI	3114.50 ± 394.94 (N = 10; 38.5%)	3134.13 ± 406.86 (N = 23; 67.6%)	.893
Abnormal maternal pregestational BMI (overweight)	3206.25 ± 428.92 (N = 8;30.8%)	3102.86 ± 459.80 (N = 7; 20.6%)	.660
Abnormal maternal pregestational BMI (obese)	3492.14 ± 438.08 (N = 8; 30.8%)	3316.25 ± 248.18 (N = 4; 11.8%)	.487
Baby femur length at 21st-23rd weeks of gestation, mm	38.39 ± 2.61 (N = 16)	39.10 ± 2.03 (N = 23)	.368
Normal maternal pregestational BMI	37.77 ± 2.17 (N = 6; 37.5%)	39.18 ± 2.05 (N = 17; 73.9%)	.177
Abnormal maternal pregestational BMI (overweight)	36.95 ± 0.87 (N = 4; 25.0%)	39.13 ± 2.73 (N = 4; 17.4%)	.180
Abnormal maternal pregestational BMI (obese)	39.98 ± 3.17 (N = 6; 37.5%)	38.45 ± 0.36 (N = 2; 8.7%)	.540

*P* values are considered statistically significant when <.05. Data are presented as mean ± SD. To describe the differences between the distribution of categorical variables and the means of the 2 groups, we used the χ^2^ test and the *t* test, respectively.

Abbreviations: BMI, body mass index; GDM, gestational diabetes mellitus.

Statistically significant difference between pregnancies with gestational diabetes mellitus (GDM) and non-GDM. Statistically significant *P* values are cited in bold

HC was greater in neonates of pregnant women with GDM compared with those of pregnant women without GDM (36.02 ± 3.49 cm and 34.48 ± 1.42 cm, respectively; *P* = .043) (Table 1). Birth length, birth weight, and baby femur length did not differ between neonates of mothers with GDM and those of mothers without. Baby femur length was increased in fetuses of GDM mothers treated with insulin compared with fetuses of GDM mothers treated by diet (Table 2). No other anthropometrical variable differed between these 2 groups.

**Table 2.** Data on GDM mothers treated with insulin or diet

Variable	Insulin (n = 13)	Diet (n = 13)	*P*
Placental weight	731.15 ± 3.13	731.27 ± 1.90	.910
Sclerostin h-score cytotrophoblasts	37.31 ± 12.68	37.27 ± 9.05	.994
Sclerostin h-score endothelial cells	7.54 ± 2.50	8.09 ± 2.17	.573
Sclerostin h-score syncytiotrophoblasts	16.38 ± 4.99	19.55 ± 2.69	.074
Sclerostin h-score stromal cells	4.08 ± 1.44	3.63 ± 1.57	.481
LRP5 hours-score cytotrophoblasts	113.46 ± 34.54	124.64 ± 30.62	.415
LRP5 hours-score endothelial cells	76.15 ± 24.59	76.36 ± 31.95	.986
LRP5 hours-score syncytiotrophoblasts	8.00 ± 4.38	11.00 ± 6.71	.201
LRP5 hours-score stromal cells	3.62 ± 1.85	4.09 ± 2.47	.595
Sclerostin umbilical	339.2 ± 453.68	101.27 ± 74.30	.119
Baby birth weight	3291.15 ± 396.39	3214.09 ± 500.82	.678
Baby length	48.46 ± 6.40	49.63 ± 1.50	.559
Head circumference	37.35 ± 4.49	34.86 ± 1.23	.090
Baby femur length	40.025 ± 2.43	36.79 ± 1.71	.012
Pregestational maternal BMI	28.340 ± 6.89	26.033 ± 2.87	.331
Maternal age	34.69 ± 5.34	33.18 ± 4.35	.461
Maternal fasting glucose	92.86 ± 26.37	87.00 ± 13.09	.692
Gestational weight gain	12.42 ± 5.41	12.09 ± 6.91	.695

Abbreviations: BMI, body mass index; h-score, histo-score; LRP5, lipoprotein receptor-related protein 5.

Among all neonates taken as a whole, none presented with macrosomia, whereas all babies had an Apgar score ≥ 8 at first minute. Body length values in all neonates correlated positively with maternal pregestational BMI and with BMI at delivery (*r* = 0.386, *P* = .002; and *r* = 0.354, *P* = .006, respectively), even after adjustment for insulin treatment (*r* = 0.818, *P* = .001; and *r* = 0.605, *P* = .029 respectively).

### Cord Blood Measurements

Umbilical glucose and umbilical sclerostin concentrations did not differ between pregnant women with and without GDM (for glucose: 75.00 ± 29.33 mg/dL vs 59.71 ± 9.32, respectively, *P* = .092; for umbilical sclerostin: 9.02 ± 14.03 pmol/L and 11.90 ± 15.87 pmol/L, respectively, *P* = .611). When all neonates were taken as a whole, umbilical sclerostin concentrations correlated positively with HC (*r* = 0.310, *P* = .049) even after adjustment for insulin treatment (*r* = 0.660; *P* = .014).

### Immunohistochemistry of Placental Sclerostin and LRP5 in Placentas From Pregnant Women With and Without GDM

**Figure 2.**
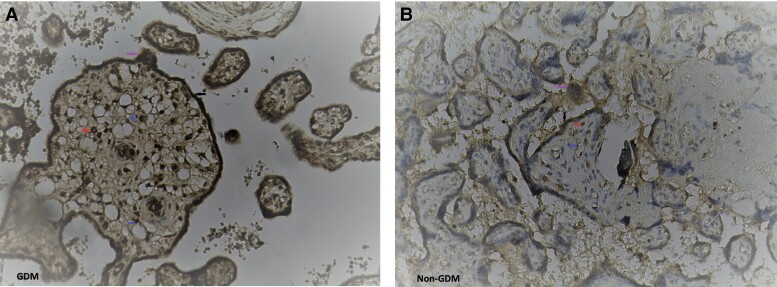
Sclerostin immunohistochemical (IHC) staining in GDM (A) and non-GDM (B) placental samples (×20). Blue arrows indicate the villous cytotrophoblasts, red arrows the villous stromal cells, purple arrows the villous endothelial cells, and black arrows the villous syncytiotrophoblasts. (A) Diffuse intense immunostaining in villous trophoblasts and in syncytiotrophoblasts, mild in endothelial cells of the villous vessels, and focally expressed in villous stromal cells. (B) Diffuse moderate immunostaining intensity of villous cytotrophoblasts and moderate in syncytiotrophoblasts, endothelial cells of the villous vessels, and villous stromal cells.

**Figure 3.**
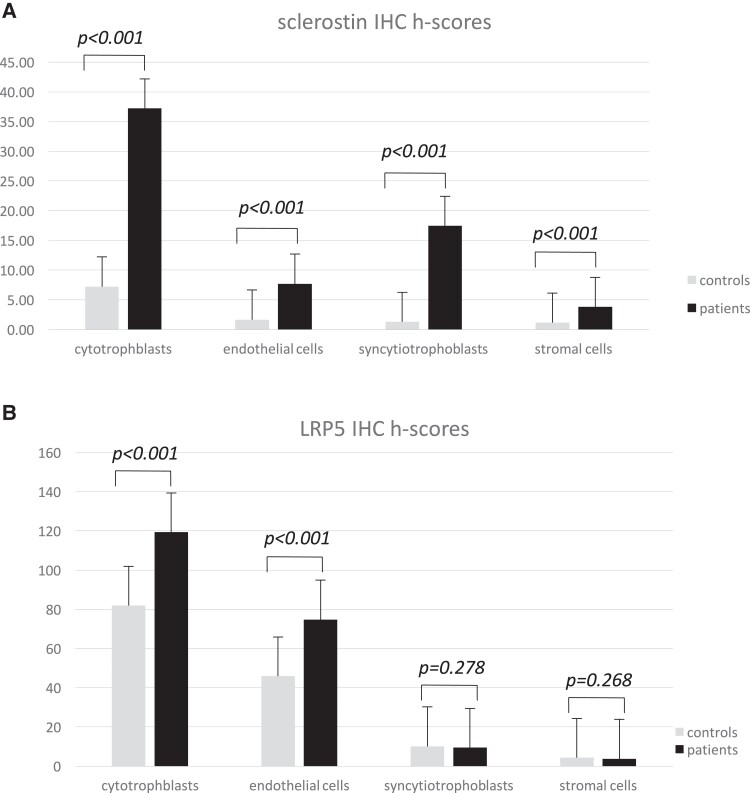
Quantitative comparisons of (A) sclerostin and (B) LRP5 IHC staining between GDM and non-GDM placentas. IHC staining was graded as: negative (0), mild (1), moderate (2), or strong (3) in the cytotrophoblasts, the endothelial cells of the villous vessels, the villous stromal cells, and the syncytiotrophoblasts. The comparisons between groups of samples were based on histo-score (h-score), which is the percentage of stained cells (labelling index) multiplied by staining intensity. h-score values are described as mean ± SD. To compare the differences between GDM and non-GDM groups, we applied Student *t* test. *P* values are considered statistically significant when <0.05.

There was no difference between GDM (731.32 ± 2.58 g) and non-GDM (731.19 ± 3.45) pregnancies regarding the weight of placentas. Immunohistochemically, placental sclerostin was mainly detected in villous regions of human term placentas. In the placentas of women with GDM, IHC was predominantly expressed in villous trophoblasts and syncytiotrophoblasts, whereas it was mild in endothelial cells of the villous vessels and focally expressed in villous stromal cells. In the placentas of women without GDM, placental sclerostin IHC expression was diffuse moderate in villous cytotrophoblasts and moderate in syncytiotrophoblasts, endothelial cells of the villous vessels, and villous stromal cells (Fig. 2). Placental sclerostin IHC expression (h-score) was significantly greater in placentas of pregnant women with GDM than in those of pregnant women without GDM, especially in villous cytotrophoblasts (h-score: 36.0 ± 12.5 and 7.35 ± 2.0, respectively; *P* < .001) and syncytiotrophoblasts (h-score: 17.2 ± 5.29 and 1.25 ± 0.44, respectively; *P* < .001) (Tables 3 and 4; Figs. 2 and 3A). Furthermore, placental sclerostin IHC expression in all placentas taken as a whole was positively correlated with pregestational maternal BMI (*r* = 0.299; *P* = .043) and HC (*r* = 0.346; *P* = .019) values and maternal fasting glucose concentrations (*r* = 0.475; *P* = .009). The IHC expression in placentas from pregnant women without GDM was weaker than in the positive controls. No correlation was found between sclerostin concentrations in umbilical serum and placental expression of sclerostin.

**Table 3.** LRP5 and sclerostin expression detected by IHC or WB

LRP5	GDM (n = 26)	Non-GDM (n = 34)	*P* adjusted
Total; h-score (IHC)	202.88 ± 58.65	145.53 ± 43.74^a^	**<**.**001**
Cytotrophoblasts; h-score (IHC)	117.15 ± 32.17	84.41 ± 28.62^a^	.**001**
Endothelial cells; h-score (IHC)	73.08 ± 28.74	46.47 ± 19.52^a^	**<**.**001**
Syncytiotrophoblasts; h-score (IHC)	8.96 ± 5.62	10.38 ± 4.43	.747
Stromal cells; h-score (IHC)	3.69 ± 2.09	4.26 ± 1.87	.607
LRP5/b-actin ratio (WB)	0.56 ± 0.12 (n = 24)	0.35 ± 0.15^a^ (n = 33)	.**009**
**Sclerostin**			
Total; h-score (IHC)	65.08 ± 17.09	11.45 ± 2.33^a^	**<**.**001**
Cytotrophoblasts; h-score (IHC)	36.0 ± 12.5	7.35 ± 2.0^a^	**<**.**001**
Endothelial cells; h-score (IHC)	7.52 ± 28.74	1.7 ± 0.47^a^	**<**.**001**
Syncytiotrophoblasts; h-score (IHC)	17.2 ± 5.29	1.25 ± 0.44^a^	**<**.**001**
Stromal cells; h-score (IHC)	3.76 ± 1.56	1.15 ± 0.37^a^	**<**.**001**
Umbilical serum sclerostin levels; pmol/L (ELISA)	9.02 ± 14.03	11.90 ± 15.87	.785

Quantitative comparisons between groups of samples were based on h-score. The observed h-scores were further analyzed according to maternal pregestational BMI and gestational weight gain. “*P* adjusted” indicates adjusted *P* value for pregestational BMI and gestational weight gain performed using univariate analysis. *P* values are considered statistically significant when < .05. Data are presented as mean ± SD. Abbreviations: BMI, body mass index; GDM, gestational diabetes mellitus; h-score, histo-score; IHC, immunohistochemistry; LRP5, lipoprotein receptor-related protein 5; WB, Western blot.

Statistically significant difference between GDM and non-GDM. Statistically significant P values are cited in bold.

**Table 4.** LRP5 and sclerostin signal intensity values and percentages of stained cells in placentas of pregnant women with and without GDM

LRP5	GDM (n = 26)	Non-GDM (n = 34)	*P*
Cytotrophoblasts; % stained cells	43.92 ± 6.98	39.26 ± 7.50	.019
Cytotrophoblasts; intensity values	2.64 ± 0.49	2.12 ± 0.48	<.001
Endothelial cells; % stained cells	27.60 ± 7.38	17.79 ± 5.92	<.001
Endothelial cells; intensity values	2.64 ± 0.49	2.53 ± 0.56	.435
Syncytiotrophoblasts; % stained cells	6.48 ± 2.29	7.91 ± 2.80	.035
Syncytiotrophoblasts; intensity values	1.36 ± 0.49	1.35 ± 0.49	.956
Stromal cells; % stained cells	3.56 ± 1.68	3.94 ± 1.63	.386
Stromal cells; intensity values	1.04 ± 0.20	1.03 ± 0.17	.828
**Sclerostin**			
Cytotrophoblasts; % stained cells	36.0 ± 12.5	7.35 ± 2.0	<.001
Endothelial cells; % stained cells	7.52 ± 28.74	1.7 ± 0.47	<.001
Syncytiotrophoblasts; % stained cells	17.2 ± 5.29	1.25 ± 0.44	<.001
Stromal cells; % stained cells	3.76 ± 1.56	1.15 ± 0.37	<.001

The intensity of staining of all placental sclerostin positive cells was mild (1) in comparison to positive control cells of human kidney. Sclerostin h-score coincides with the percentage of sclerostin-positive stained cells.

Abbreviations: GDM, gestational diabetes mellitus; LRP5, lipoprotein receptor-related protein 5.

**Figure 4.**
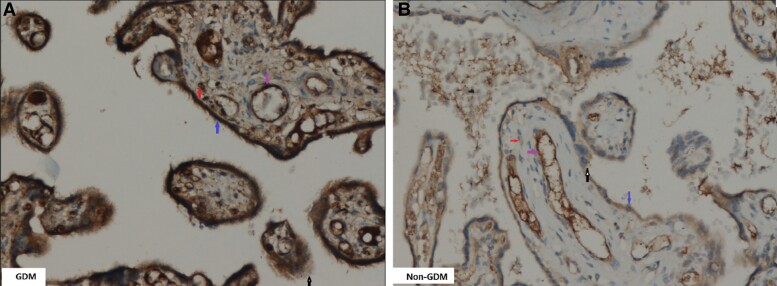
LRP5 immunohistochemical (IHC) staining in GDM (A) and non-GDM (B) placental samples (×20). Blue arrows indicate the villous cytotrophoblasts, red arrows the villous stromal cells, purple arrows the villous endothelial cells, and black arrows the villous syncytiotrophoblasts. (A) Diffuse intense immunostaining of villous cytotrophoblasts and endothelial cells of the villous vessels, moderate in villous syncytiotrophoblasts, and focally mild in villous stromal cells. (B) Diffuse moderate immunostaining in villous cytotrophoblasts and villous endothelial cells, mild in villous syncytiotrophoblasts, and focally mild in villous stromal cells.

Immunohistochemically, the LRP5 protein was mainly detectable in villous regions of human term placenta. In the placental tissues, IHC expression of LRP5 was found predominantly in villous cytotrophoblasts and in endothelial cells of the villous vessels and focally in villous stromal cells and syncytiotrophoblasts (Fig. 4). Furthermore, LRP5 IHC h-score and intensity correlated negatively with placental weight (*P* = .021, *r* = −0.302; *P* = .003, *r* = −0.384; respectively). IHC of LRP5 was significantly greater in villous cytotrophoblasts (h-score: 117.15 ± 32.17 and 84.41 ± 28.62, respectively; *P* < .001) and in endothelial cells of the villous vessels (h-score: 73.08 ± 28.74 and 46.47 ± 19.52, respectively; *P* < .001) of the placentas of pregnant women with GDM than in those without GDM (Tables 3 and 4; Figs. 3B and 4). IHC expression of LRP5 in villous cytotrophoblasts, endothelial cells of the villous vessels, villous stromal cells, and in syncytiotrophoblasts in placentas from women without GDM was weaker than in the positive control. In addition, the IHC expression of placental sclerostin in all placentas taken as a whole correlated positively with that of LRP5 (*r* = 0.529; *P* < .001). Finally, the expression of placental sclerostin and LRP5 did not differ in placentas of pregnant women with GDM between either those treated by insulin or those treated by diet (Table 2). Of note, according to the statistically significant comparisons included in Table 2 (Results), the observed power analysis of the present pilot study was found to be >0.90.

### Western Blotting of LRP5 in Placentas From Pregnant Women With and Without GDM

**Figure 5.**
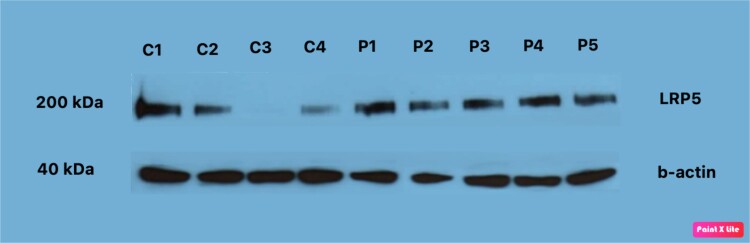
(A) Representative Western blot analysis and (B) ratio LRP5/b-actin levels in GDM and non-GDM placental tissues. C1, C2, and C3 indicate 3 different non-GDM samples; P1, P2, P3, and P4 indicate 4 different GDM samples. Values are presented as mean ± SD (22 samples/32 controls, *P* < .001).

Greater LRP5 protein expression levels were detected by Western blotting analysis in pregnant women with GDM compared with those without GDM (*P* < .001) (Fig. 5; Table 3). Expression of LRP5 by Western blotting did not differ in placentas of pregnant women with GDM between either those treated by insulin or those treated by diet (data not shown). When all women were taken as a whole, LRP5 levels by Western blotting were significantly correlated with immunohistochemical LRP5 levels (*r* = 0.324; *P* = .016). When all pregnant women were taken as a whole, no correlation was found between LRP5 immunohistochemical or Western blotting levels and anthropometric values or glucose concentrations.

## Discussion

In the present study, we detected, for the first time to our knowledge, the presence of placental sclerostin and LRP5 in the maternal side of placental tissue derived from women with and without GDM. Placental sclerostin was mainly detected in villous cytotrophoblasts, whereas LRP5 was predominantly detected in villous cytotrophoblasts as well as in endothelial cells of the villous vessels of the placenta. Sclerostin is an osteokine produced predominantly by osteocytes that antagonizes LRP5 in bone metabolism, both molecules being part of the canonical Wnt-signalling pathway (31). Results from human and animal studies have shown that the Wnt-signalling pathway interacts with insulin secretion and action posing intriguing questions on the role of sclerostin and LRP5 in other molecular circuits and in tissues other than bone (14, 15, 20-22, 32, 33).

Furthermore, in the present study, placental sclerostin and LRP5 expression in women with GDM was significantly greater than in pregnant women without GDM, whereas placental sclerostin expression correlated positively with second-trimester maternal fasting glucose concentrations. Interestingly, a similar up-regulation of sclerostin has been demonstrated in the tibia of insulin resistant rats associated with increased mRNA levels of *Lrp5*, an activator of the Wnt-signalling pathway (16). Studies on *Lrp5*−/− mice showed a markedly impaired glucose tolerance, whereas glucose-induced insulin secretion in these mice was decreased, suggesting that a functional LRP5 is required for normal glucose metabolism (14). In a recent study in cultured mouse mammary epithelial cells, loss of *Lrp5* led to profound growth suppression that, in association with limited external glucose administration, induced mitochondrial stress, suggesting a Wnt-independent role of Lrp5 in glucose uptake as well (34). Recently, a gain-of-function *Lrp5* mutation in insulin-deficient mice was shown to fully protect bone mass and strength in prolonged hyperglycemia, to improve peripheral glucose metabolism, and to prevent whitening of brown adipose tissue (15). Other authors have shown that LRP5 gain-of-function mutations do not affect glucose metabolism (35). In GDM, pancreatic insulin secretion does not suffice to meet insulin demands, whereas derangements of several placental signaling pathways have been described (26, 27, 36, 37). The observed overexpression in the placental sclerostin/LRP5 pathway in the studied GDM placentas might represent the development of an adaptive mechanism in face of maternal hyperglycemia. Of note, in the present study, expression of both molecules in placentas of pregnant women with GDM did not differ between those treated either by insulin or by diet. This observation may suggest that placental sclerostin and LRP5 are overexpressed in GDM via insulin-independent mechanisms, as detected in mice (15).

In addition, in the present study, both in human uncomplicated and GDM pregnancies, placental sclerostin expression correlated positively with pregestational maternal BMI values. In the past, maternal sclerostin concentrations, in peripheral circulation, were correlated positively with pregestational BMI of uncomplicated human pregnancies (22). Positive associations between serum sclerostin concentrations and BMI have also been recorded in children and adolescents (38). Moreover, in a recent study, serum maternal sclerostin concentrations were correlated with ultrasound fetal intra-abdominal fat measurements, suggesting a possible endocrine involvement of maternal sclerostin into fetal intra-abdominal adipose tissue deposition during pregnancy (23). Of note, in mice, sclerostin has been found to inhibit Wnt-signalling pathway in preadipocytes and thus to promote adipose tissue formation in bone marrow (39).

On the other hand, in vitro and in vivo studies suggest that the Wnt-signalling pathway contributes to the development of early trophoblasts and their differentiation and invasion (19). Thus, abnormal activation of the Wnt/β-catenin signalling pathway may lead to trophoblast disorders such as choriocarcinoma or preeclampsia (40). By analogy to these reports, it is possible that, in pregnant women with GDM, overexpression of placental sclerostin and LRP5 may reflect hyperactivation of the Wnt/b-catenin signalling pathway resulting in altered placenta development.

Ιn the present study, information regarding newborns’ anthropometrics was investigated and reported for showcasing that, adverse outcomes in newborns’ health, often associated with untreated GDM (ie, macrosomia), have not been observed. Furthermore, umbilical sclerostin concentrations, which reflect neonatal metabolism and bone mineral content (s), correlated positively with neonatal head circumference.

A limitation of this study is that the reported expression of placental sclerostin and LRP5 was not followed by the analysis of other downstream molecules in the Wnt-signalling pathway. This option is part of future projects. In any case, this is the first time that both LRP5 and sclerostin are investigated in placenta and umbilical blood, in a cohort of GDM and non-GDM pregnant women whose delivery outcomes are recorded. Other limitations of this study are that the GDM population was not homogenous regarding the therapeutic control of hyperglycemia (half under insulin treatment and half under diet); insulin and c-peptide concentrations were not measured in cord blood; and data on physical activity and on prepregnancy diet were not collected. It is important to highlight that the present study is small, demonstrating cross-sectionally an association that, for clinical data, relies on medical record data. Replication of these data is necessary to confirm their scientific strength.

In conclusion, placental sclerostin and LRP5 were detected in the villous region of human placentas and their expression was increased in GDM compared with non-GDM placentas. Although a direct involvement of sclerostin and LRP5 in the causality of GDM is not certain, it is possible that these molecules might interfere with the direct and/or indirect improvement of glucose metabolism. The contribution of the Wnt/LRP5/sclerostin signalling pathway in glucose metabolism warrants further study as the recent in vitro findings suggest new perspectives for therapeutic targets in humans regarding GDM.

## Data Availability

Original data generated and analyzed during this study are included in this published article or in the data repositories listed in References.

## Abbreviations

BMI: body mass index
GDM: gestational diabetes mellitus
gwk: gestational week
HC: head circumference
h-score: histo-score
IHC: immunohistochemistry
LRP5: low-density lipoprotein receptor-related protein 5
OGTT: glucose tolerance test
PBST: PBS with Tween
T2DM: type 2 diabetes mellitus
WB: Western blotting
